# Erythema Multiforme in an HIV+ Patient on Highly Active Antiretroviral Therapy After Starting Paxlovid (Nirmatrelvir-Ritonivir)

**DOI:** 10.7759/cureus.56487

**Published:** 2024-03-19

**Authors:** Alison D Cáceres, Kristy Bono, Neil Kothari

**Affiliations:** 1 Internal Medicine, Rutgers University New Jersey Medical School, Newark, USA; 2 Medicine, Rutgers University New Jersey Medical School, Newark, USA

**Keywords:** medication interaction, covid-19, hiv positive, nirmatrelvir-ritonivir, paxlovid, erythema multiforme

## Abstract

In this report, we present a case of a woman currently on HIV antiretroviral therapy who presented with oral mucosal and cutaneous skin lesions with a target-like appearance following completion of a five-day course of Paxlovid™ for symptomatic COVID-19 infection. The patient was treated with intravenous steroids and oral antihistamines with mild improvement. However, she returned in one week with worsening skin lesions. The biopsy and infectious workup were non-contributory. It was determined that the patient had developed erythema multiforme (EM), secondary to Paxlovid™.

## Introduction

A variety of factors can induce erythema multiforme (EM), a rare, acute, immune-mediated mucocutaneous response. Most commonly, it can occur following a Herpes simplex virus (HSV) type 1 infection and is less commonly precipitated by the use of certain medications such as nonsteroidal anti-inflammatory drugs (NSAIDs), sulfonamides, antiepileptics, and antibiotics (<10%) [1,2]. Generally, EM can present in either of two variants: major, typically involving multiple mucous membranes (not limited to the oral cavity, genital, ocular, laryngeal, or oesophageal mucosa), and minor, affecting only one mucosa and appearing as the classic target-like cutaneous lesions [1].

The frequency of mucosal involvement has been estimated at 25-60%, most commonly appearing in the lips and anterior mouth 70% of the time. These oral mucosal lesions often appear in multiples and are large, shallow, and extremely painful ulcerations [2]. They initially present as erythema and edema, progressing into superficial erosions with pseudomembranous formations [3]. However, the clinical presentation of EM may vary, making diagnosis difficult. Oral biopsies of EM typically exclude other more prominent causes, like HSV, making the histological findings non-diagnostic [2]. Rarely, these oral biopsies show epithelial edema and acanthosis, sometimes with an irregular elongation of rete ridges, and in the connective tissue, a combination of vascular dilatation and congestion, perivascular infiltrate of mononuclear cells, and edema of the upper portion of the lamina propria [3,4]. While there are no specific diagnostic laboratory tests, EM lesions are usually diagnosed clinically. 

Paxlovid™ was the first outpatient oral pharmacological therapy approved by the Food and Drug Administration for COVID-19 infection [5]. It is a combination protease inhibitor of nirmatrelvir and ritonivir. Nirmatrelvir inhibits the SARS-CoV-2 protease, while ritonavir inhibits CYP3A, allowing the enhancement of nirmatrelvir [6]. The medication has been shown to significantly reduce hospitalization and death rates among high-risk patients with COVID-19 infection [7]. Given the widespread use of this drug, it is important for clinicians to be aware of all potential side effects, such as maculopapular rash, hyperkeratosis, hyperhidrosis, pruritus, skin edema, skin exfoliation, and alopecia, as well as the possibility of adverse drug interactions with existing medications that patients may be taking.

## Case presentation

A 64-year-old woman with HIV infection on highly active antiretroviral therapy (HAART) presented to the emergency department in early February 2023 with progressive painful oral mucosal ulcerations that developed after completing a five-day course of Paxlovid™ (nirmatrelvir-ritonavir) for a recent COVID-19 infection following a positive at-home COVID-19 test. The patient originally presented to the emergency room in early February with one day of bilateral eye itchiness and discharge, lip swelling, and mouth cavity ulcers. She reported just completing a five-day treatment of Paxlovid™ following a positive at-home COVID test at the end of January. Additionally, she reported receiving COVID-19 vaccines at least four times on August 19, 2021, April 4, 2022, September 15, 2022, and October 11, 2022, without any reported adverse reactions to COVID-19 vaccines or any other vaccines in the past. Further history revealed an allergy to erythromycin, diagnosed four months ago when prescribed for an eye infection, with the subsequent development of increased eye redness, lip swelling, and nasal congestion. She also reports a reaction to a hair removal product that her sexual partner used two months ago. She reports developing lip redness and swelling after her lips were in contact with her partner's skin.

The patient was subsequently admitted for one day for observation and treated with intravenous steroids and oral antihistamines due to a presumed allergic reaction to Paxlovid™. She was discharged the next day once mild improvements in her symptoms were appreciated. 

**Figure 1:**
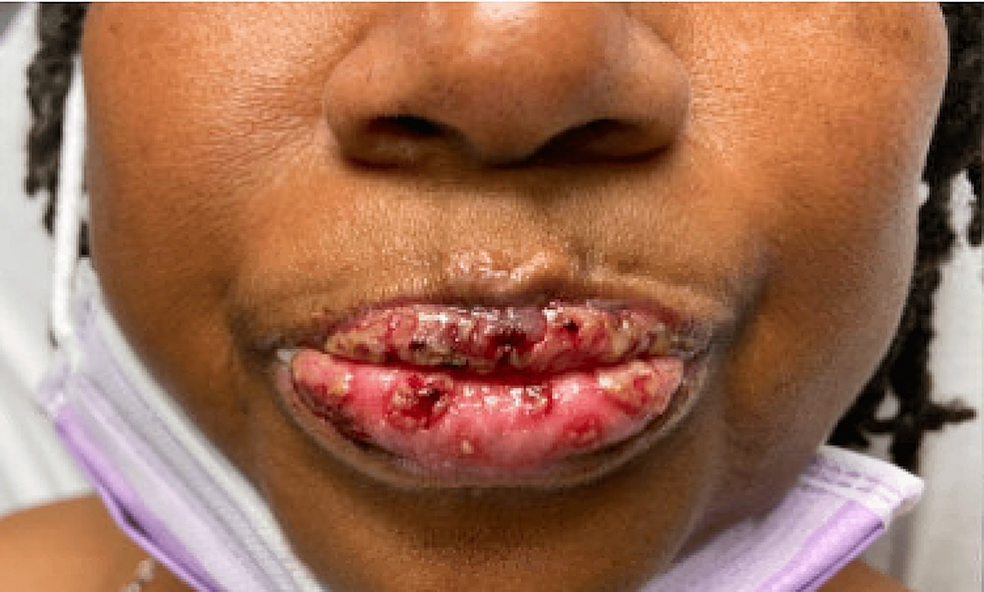
Patient’s ulcerated lips.

**Figure 2:**
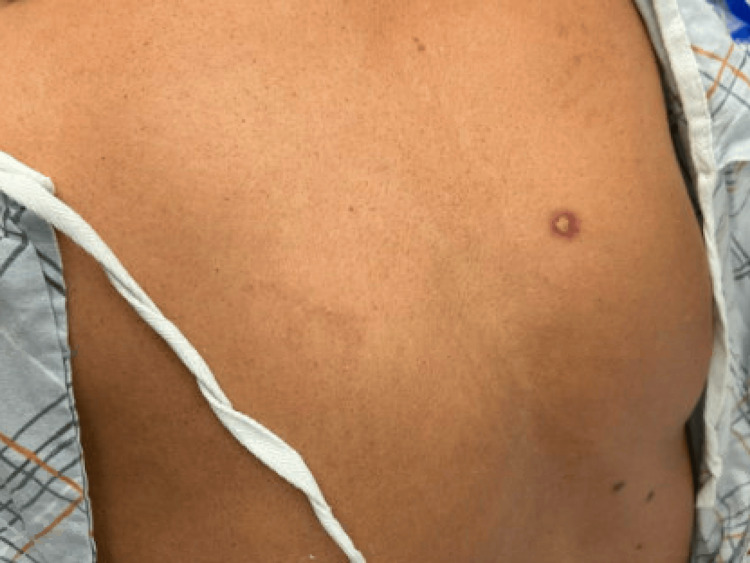
Single target-like lesion on mid right back.

**Figure 3:**
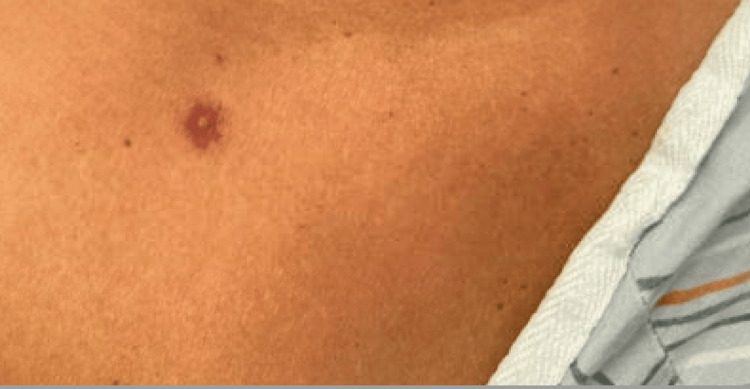
Single target-like lesion on upper right chest.

However, one week later, the patient presented to the emergency room once again with a significant worsening of her system. At this time, she complained of bilateral eye pruritus, ocular discharge, crusting mucosal swelling of the lips, oral cavity pruritus, oral and nasal ulcers, odynophagia, and a rash localized on her back. She did not mention any involvement of the gastrointestinal mucosa. At home, she was applying petroleum jelly and an over-the-counter topical cortisone to her lips with only minimal symptomatic relief. Her HAART consisted of Tivicay (dolutegravir) and Symtuza (darunavir/cobicistat/emtricitabine/tenofovir alafenamide); she reported no changes to this regimen over the last 18 months. On physical examination, the patient demonstrated increased crusting around both eyes and diffuse ulcers on both lips, in her oral cavity, on her tongue, and in her bilateral nares, as shown in Figure 1. In addition, a few scattered skin lesions with a target-like appearance were also noted on our patient’s chin, upper chest, and mid-back, as seen in Figures 2-3.

The patient’s most recent HIV serology demonstrated a CD4+ count of approximately 2,061 cells/µl and a viral load of less than 20 cp/mL. Her white blood cell count was 7.7 × 10^3/µl, and her electrolytes were within normal limits.

The patient was re-admitted to the medical service for observation. A flexible fiberoptic laryngoscopy exam revealed ulceration of the septum and turbinates bilaterally, ulceration of the posterior and lateral pharyngeal walls, and diffuse mild edema and erythema without airway obstruction. The ophthalmic evaluation was unremarkable, as conjunctiva/fornices were clear, the cornea was clear, and there was no further discharge/drainage. She was started on IV acyclovir, pending a viral workup. In addition, topical steroids (clobetasol) and oral viscous lidocaine were initiated to reduce oral pain. Her inflammatory workup subsequently revealed negative double-stranded DNA and C-reactive protein levels of 6 mg/L. For infectious workup from blood samples, results showed non-reactive rapid plasma reagin, negative cytomegalovirus (CMV) quantitative by PCR, and negative CMV IgM by ELISA. She tested positive for CMV IgG, HSV-1 IgG, and HSV-2 IgG. The patient’s nasal swab was negative for influenza A, influenza B, respiratory syncytial virus, and SARS-CoV-2 RNA by PCR. A biopsy of her buccal mucosa was performed on hospital day #2 and showed unremarkable squamous mucosa with mild lymphocytic infiltration. The biopsy was negative for immunofluorescence to the antibodies IgG, IgM, IgA, and C3. Cultures from the buccal mucosa were negative for HSV-1 and HSV-2 by PCR. As the patient felt symptomatically improved with decreased pain and odynophagia, she was discharged to home with close outpatient follow-up by the allergy/immunology service. Due to this patient's medical history and documented allergic reactions, she was recommended to avoid Paxlovid™ in the future, warranting a re-challenge to prevent serious COVID-19 complications in the future. At a follow-up visit several weeks later, her lesions were noted to have fully resolved.

## Discussion

Our brief report describes the development of EM after completion of Paxlovid™ for COVID-19 infection. To date, there have been several adverse cutaneous manifestations of this drug that have been documented, including maculopapular rash, hyperkeratosis, hyperhidrosis, pruritus, skin edema, skin exfoliation, and alopecia [8]. However, there have been very few reports documenting the development of EM related to the use of this medication, especially due to drug interactions [9]. Conversely, EM has been documented as a side effect of other possible COVID-19 treatments, such as hydroxychloroquine and lopinavir-ritonavir [10-12]. This case report suggests that Paxlovid™ should also be added to this list. 

There are other possible causes of EM in this patient to explore. To start, infectious causes, such as Kawasaki disease or syndrome, have been associated with COVID-19 and drug use (nirmarelvir/ritonavir) and similarly present with ulcerated lesions of the lips, oral and nasal mucosa, skin rash on the trunk, and conjunctival lesions. While the literature states this is usually seen in children, it should be considered among the diagnostic possibilities [13]. Of note, literature has documented the development of EM in patients using HAART therapy [14]. It is possible that our patient developed EM due to a drug interaction between the COVID-19 treatment and HAART medications, as ritonavir, dolutegravir, darunavir, and cobicistat are all known CYP3A inhibitors [15-17]. Thus, this combination of drugs could have caused a hypersensitivity reaction due to an increase in serum levels of any of these drugs. It is also possible that the rash is secondary to the COVID-19 infection itself, as prior studies have suggested that EM may form weeks after the start of the COVID-19 infection [18]. Similarly, this patient’s EM reaction occurred after the completion of Paxlovid™ and after the first admission. COVID testing was negative during the second admission. Specifically, one study found the mean length of time was 19.5 days from the onset of COVID-19 symptoms, while this patient rapidly developed symptoms following the completion of Paxlovid™ treatment [19].

Overall, this case highlights the importance of careful drug consideration for immunocompromised patients. People infected with HIV are at significantly higher risk of cutaneous drug reactions [20]. Moreover, patients on HAART therapy may be predisposed to increased drug reactions due to the administration of multiple protease inhibitors. It is important for healthcare providers to be aware of potential risk factors for the development of adverse effects from Paxlovid™.

## Conclusions

Paxlovid™ (nirmatrelvir-ritonivir) has limited documented cutaneous side effects. With its wider usage for the treatment of COVID-19, there is increasing significance in understanding its potential side effect profile. This case report demonstrates the development of EM in an HIV patient on HAART, emphasizing the importance of considering the possibility of infectious causes and negative drug interactions, especially when patients are on multiple protease inhibitors.
